# HOW DOES THE NEIGHBORHOOD-BUILT ENVIRONMENT CONTRIBUTE TO SOCIAL HEALTH FOR PEOPLE LIVING WITH DEMENTIA?

**DOI:** 10.1093/geroni/igae098.2045

**Published:** 2024-12-31

**Authors:** Janissa Altona, Emily Mena, Benjamin Schüz, Karin Wolf-Ostermann

**Affiliations:** Institute of Public Health and Nursing Research, Bremen, Bremen, Germany; University of Bremen, Bremen, Bremen, Germany; University of Bremen, Bremen, Bremen, Germany; University of Bremen, Bremen, Bremen, Germany

## Abstract

Several studies point to the importance of the neighbourhood-built environment (NBE) for the social health of community-dwelling people living with dementia (PlwD). Indicators of dementia-friendly urban planning can be identified in the literature, but little is known about which specific factors of the NBE are important for PlwD and family carers. In order to examine relevant indicators, the following questions were analyzed by a qualitative research approach in the German DEN-HB study: 1. Which places in the residential and living environment are subjectively perceived as positive or negative by PlwD and their carers, and how satisfied are they with the dementia-friendliness of their social space? 2. What is the role of social aspects of the NBE and which aspects are conducive to social health? Based on a systematic review of NBE factors and a subsequent empirical study on evaluating existing wayfinding structures in the federal state of Bremen, Germany, we will conduct individual interviews with PlwD and their informal carers to discuss the relevance of these factors. Additionally, a focus group with dementia coordination centres and responsible persons from ministerial institutions in Bremen, Germany, will be conducted to discuss possible actions in urban planning. Results of both qualitative research approaches will be presented and discussed to promote participatory approaches to the design of the NBE for PlwD and, in particular, to focus more strongly on social health factors.

